# A review of creatine supplementation in age-related diseases: more than a supplement for athletes

**DOI:** 10.12688/f1000research.5218.1

**Published:** 2014-09-15

**Authors:** Rachel N. Smith, Amruta S. Agharkar, Eric B. Gonzales

**Affiliations:** 1 Department of Pharmacology & Neuroscience, UNT Health Science Center, Fort Worth, TX, TX, 76107, USA; 2 Institute for Aging and Alzheimer’s Disease Research, UNT Health Science Center, Fort Worth, TX, TX, 76107, USA; 3 Cardiovascular Research Institute, UNT Health Science Center, Fort Worth, TX, TX, 76107, USA

**Keywords:** Creatine, central nervous system, age-related diseases

## Abstract

Creatine is an endogenous compound synthesized from arginine, glycine and methionine. This dietary supplement can be acquired from food sources such as meat and fish, along with athlete supplement powders. Since the majority of creatine is stored in skeletal muscle, dietary creatine supplementation has traditionally been important for athletes and bodybuilders to increase the power, strength, and mass of the skeletal muscle. However, new uses for creatine have emerged suggesting that it may be important in preventing or delaying the onset of neurodegenerative diseases associated with aging. On average, 30% of muscle mass is lost by age 80, while muscular weakness remains a vital cause for loss of independence in the elderly population. In light of these new roles of creatine, the dietary supplement’s usage has been studied to determine its efficacy in treating congestive heart failure, gyrate atrophy, insulin insensitivity, cancer, and high cholesterol. In relation to the brain, creatine has been shown to have antioxidant properties, reduce mental fatigue, protect the brain from neurotoxicity, and improve facets/components of neurological disorders like depression and bipolar disorder. The combination of these benefits has made creatine a leading candidate in the fight against age-related diseases, such as Parkinson’s disease, Huntington’s disease, amyotrophic lateral sclerosis, long-term memory impairments associated with the progression of Alzheimer’s disease, and stroke. In this review, we explore the normal mechanisms by which creatine is produced and its necessary physiology, while paying special attention to the importance of creatine supplementation in improving diseases and disorders associated with brain aging and outlining the clinical trials involving creatine to treat these diseases.

## Introduction

The usage of dietary supplementation in the United States is a multibillion-dollar industry, where creatine ( *N*-[aminoiminomethyl]- *N*-methyl glycine) accounts for over 4 million kg and $200 million annually ^1, 2^. Creatine is an endogenous molecule found in all cells in the body and is synthesized in the kidney, liver, and pancreas using the amino acids arginine, glycine and methionine before entering the bloodstream ^3– 5^. From the plasma, creatine is transported into the cells via the creatine transporter protein (CRT) ^6, 7^. This transporter is critical for the distribution of creatine throughout the cells as well as for traversing the blood brain barrier (BBB), giving creatine access to the central nervous system (CNS).

Nearly 95% of creatine stores reside in skeletal muscle with the remaining 5% found in the brain, liver, testes, and kidneys ^8^. Perhaps the most well understood role of creatine in physiology is its participation in energy production. More specifically, creatine maintains the intracellular levels of adenosine triphosphate (ATP) in skeletal muscle. This source of ATP is produced via oxidative phosphorylation, which is regulated by the mitochondria ^9^. Within just a few seconds, muscle contraction utilizes the entire ATP store (2–5 mM) found in skeletal muscle ^10^. ATP is regenerated using the phosphocreatine system where phosphocreatine donates its phosphate group to adenosine diphosphate (ADP) to form ATP. This reaction occurs rapidly and reversibly via the enzyme creatine kinase (CK), making the ATP replenishing capacity of both phosphocreatine and creatine kinase high. Conversely, at rest, ATP donates a phosphate group to creatine in order to replenish phosphocreatine stores for future muscle contraction use.

Although primarily associated with energy production, mitochondria play an important role in the production of reactive oxygen species, dysregulation of calcium, excitotoxicity, and premature cellular death ^11– 14^. Likewise, creatine has important implications in antioxidant mechanisms, controlling intracellular calcium concentrations, regulating extracellular glutamate concentrations, and preventing the opening of the mitochondrial permeability transition pore (MPT) ^15– 20^. With evidence for creatine’s critical role in cellular bioenergetics, the phosphocreatine system in energy buffering, and the aforementioned implications in mechanisms associated with mitochondrial dysregulation, it is no surprising that creatine is the subject of investigation for improving the status of patients with neurodegenerative diseases that either result or progress by some mechanism of energy insufficiency.

## Physiological creatine concentrations and creatine supplementation

There is a maximum capacity for the synthesis of endogenous creatine. To increase these levels, patients and athletes turn to creatine supplementation. These individuals that take in foods rich in creatine tend to have higher creatine levels ^21, 22^. The transport of creatine into cells is limited, since the capacity of creatine transport within each muscle cell is only 160 mmol/kg ^23^. The possible beneficial effects of creatine are negligible if the creatine transporter is not functioning or if the maximal concentration of creatine within the cell has been reached. Evidence suggests that additives with creatine supplementation like proteins, carbohydrates, alpha lipoic acid, and D-pinitol can stimulate the movement of creatine into the cell, making creatine an ideal supplement for athletes with increased protein and carbohydrate intake ^24– 27^.

In general, a 70 kg human has a total creatine pool of 120 grams with 2 grams per day production from both dietary and endogenous sources ^8, 28^. Like many other supplements, supplementation reduces the normal physiological creatine production. This reduction is reversible as creatine supplementation is terminated ^8^. Athletes use creatine supplementation to increase creatine phosphate stores. Elevated phosphocreatine leads to the phosphorylation of ADP to ATP and aids in limiting energy depletion during rapid muscle movement. Multiple studies have indicated significant improvements in sprint performance, body mass, fat-free body mass, weightlifting volumes, oxygen uptake and overall exercise performance following creatine supplementation ^29– 40^. Creatine loading in athletes can require 20 grams per day of supplementation, while maintenance dosing is roughly 5 grams per day. These studies dosed the subjects in a similar fashion. Serum creatine levels reached 2.17 mM and 0.8 mM at 2.5 hours following a 20 gram and 5 gram creatine bolus, respectively ^41, 42^. Creatine is excreted in the urine as creatinine with a daily turnover of 2 grams per day. Although creatine supplementation results in reduced, but reversible natural creatine production, creatine supplementation appears to have few unwanted side effects ^43^. Thus, creatine is an attractive dietary supplement for athletes.

Prior to the usage of creatine as an athletic enhancer, creatine has been the focus of research to understand the dietary supplement’s role in physiology for 150 years. Creatine supplementation became popular during the Barcelona Olympic Games as it was shown to enhance athletic performance ^44^. Around the same time, two studies showed that creatine enhanced exercise performance via oral creatine ingestion ^23, 45^. With a clear understanding of the creatine/phosphocreatine system and its relation to the ADP/ATP energy metabolism in the mitochondria, studies began to shift their focus to understanding creatine’s role in pathophysiological conditions.

In addition to athletic performance, creatine usage has expanded to treat pathophysiological conditions including gyrate atrophy, post-stroke depression, congestive heart failure, chronic musculoskeletal pain disorders, atherosclerotic diseases and cisplatin nephrotoxicity ^28, 46, 47^. Furthermore, a recent review proposed prophylactic creatine supplementation could reduce chances of preterm labor or hypoxic-ischemic encephalopathy ^48^. Extensive research has demonstrated that the availability of phosphocreatine plays a role in skeletal muscle pathology and the associated pain can be alleviated by the intake of exogenous creatine ^49^. New studies indicate that creatine plays a role in age-related neurological diseases and reduced brain functionality associated with Parkinson’s disease, Huntington’s disease, amyotrophic lateral sclerosis (ALS), long-term memory deficits, Alzheimer’s disease, and stroke. In the subsequent text, we will discuss creatine’s role in these neurodegenerative conditions.

## Benefits of creatine supplementation in age - associated declines in the brain

### General benefits

Aging is associated with lower levels of creatine and phosphocreatine, specifically in the skeletal muscle. Phosphocreatine regeneration rates following exercise fall approximately 8% each decade after age 30 ^50^. Creatine supplementation increases both creatine and phosphocreatine from 10–40% in athletes ^50^. Furthermore, a recent review described a meta-analysis of the role creatine supplementation and resistance training plays on muscle health in an aging population. Based on the analysis of 13 published, creatine had an overall beneficial effect on aged individuals muscle mass ^51^. Since the creatine transporter can readily transport creatine from the bloodstream across the BBB, it is reasonable to suggest that exogenous supplementation of creatine would increase concentrations in the brain, where endogenous creatine levels may be diminished as a person ages. In neurodegenerative disorders (as outlined below), creatine may help slow the progression of each condition.

### Parkinson’s disease

Parkinson’s disease (PD) is a neurodegenerative disorder resulting from the loss of dopamine neurons in the midbrain with symptoms becoming apparent when approximately 60% of these neurons are lost ^52^. Notable symptoms of PD include resting tremor, postural instability, bradykinesia, loss of muscle mass, strength, and increased ability to fatigue. The treatment measures for PD involve early detection of the disease and understanding how to slow PD progression once symptoms have been reported. Rodent models often used for the study of PD are induced by toxins, such as 1-methyl-4-phenyl-1,2,3,6-tetrahydropyridine (MPTP), to mimic the pathogenesis and progression of the disease marked by dopaminergic neuron loss, mitochondrial dysfunction, and oxidative stress ^53, 54^. In particular, complex I of the electron transport chain within the mitochondria is deficient in patients with Parkinson’s disease. The fact that postural instability, loss of muscle mass, and strength all occur in the progression of PD, and are coupled with creatine’s ability to alter cellular energetics, has led to the hypothesis that the creatine dietary supplementation could minimize the associated symptoms with Parkinson’s disease.

An early study testing the benefit of creatine in MPTP-induced Parkinson’s disease mice showed significant neuroprotection with 1% creatine supplementation in diet ^55^. In 2006, a group of investigators at the National Institute of Neurological Disorders and Stroke (NINDS) began a phase III clinical trial for creatine in 200 patients affected by Parkinson’s disease after second phase preliminary data showed that creatine was able to slow down the progression of the disease ^56^. A year later, a double-blind study compared the control group (no creatine supplementation) to the test group (20 Parkinson’s disease patients), which received a creatine loading dose of 20 grams per day for 5 days and a creatine maintenance dose of 5 grams per day thereafter ^57^. The purpose of this study was to specifically explore if creatine could help increase muscle strength in idiopathic Parkinson’s disease patients. Both groups received resistance training during the study. A difference was observed in the creatine supplemented group with some of the strength exercises used versus the control group ^57^. In September 2013, the NINDS announced that the phase III clinical trial for creatine use in Parkinson’s disease was halted because the study would result in an observable significant difference ^58^. The test subjects tolerance for creatine or creatine associated side effects were not the cause for stopping the trial. The patients in this creatine clinical trial received 10 grams creatine daily for up to 5 years. Although this outcome is disappointing, the ability for creatine to alter energy dysfunction in addition to muscle strength may still have a combinatory effect with other Parkinson’s disease drug treatments. The possibility remains that creatine may be beneficial for Parkinson’s disease patients, but more work needs to be done to demonstrate the dietary supplements efficacy.

### Huntington’s disease

Huntington’s disease (HD) is a neurodegenerative disease where the onset of symptoms occurs in midlife. Once the symptoms begin, a patient can expect to live, on average, 20 more years ^59^. Those that develop Huntington’s disease possess genetically inherited mutations in the number of cytosine-adenine-guanine (CAG) repeats in the *huntingtin* gene responsible for producing the huntingtin protein ^60– 63^. The huntingtin protein is expressed throughout the central and peripheral nervous systems. Upon the onset of HD symptoms, the patient begins to exhibit changes in mood, cognition, and motor coordination ^60– 63^. In addition to contributing to an abnormal gait, resting tremor and even epileptic seizures associated with Huntington’s disease, the mutant huntingtin protein product leads to impaired energy metabolism ^64^. Without a cure for Huntington’s disease, the impairment of energy metabolism offers an avenue and target for new therapies. Furthermore, there is an observed reduction in the phosphocreatine and inorganic phosphate ratio in Huntington’s disease patients’ muscle tissue, which may indicate the Huntington mutation’s involvement in dysregulating the phosphocreatine/creatine ratio ^65^. In a mitochondrial toxin Huntington’s disease mutant mouse model, R6/2, creatine is hypothesized to act as a means of buffering, or providing a larger phosphocreatine pool for rapid conversion of ADP to ATP, energy within the cell ^5, 66– 68^. Furthermore, as an extension of the work done by Matthews and colleagues ^68^, the Ferrante group ^69^ showed that lifespan was extended by 9.4, 17.7 and 4.4% when supplemented with 1, 2, or 3% creatine in the diet, respectively ^69^.

Several Huntington’s disease transgenic mice strains have been developed to study the dysfunction in energy metabolism, the electron transport enzymes in the mitochondria, and excessive excitotoxicity associated with the disease ^53, 70– 74^. Supplementation with exogenous creatine in one transgenic mouse model showed improved motor performance, reduced atrophy of neurons, and huntingtin protein aggregates, and an observed increased survival rate or life-span ^17, 69, 75^. Following these studies, a phase II clinical trial ensued to assess creatine tolerability given at a dosage of 8 grams per day to Huntington’s disease patients ^76^. From these studies, Hersch and colleagues determined that 8-hydroxy-2’-deoxyguanosine (8OH2’dG), a marker for damaged DNA, was abnormally high in patients with HD, but was reduced after creatine treatment ^76^. In an interview regarding the phase III clinical trial of creatine usage in Huntington’s disease patients, the CREST-E (Creatine Safety, Tolerability, and Efficacy) clinical trial, Hersch reported that 8OH2’dG had returned to normal levels. Subsequently, there was an observed reduction in brain deterioration rate when patients were supplemented with creatine and that creatine kinase is a potential biomarker for HD ^77^. The potential of creatine as a viable therapy for HD remains to be seen and the results from CREST-E clinical trial will provide some indication to the dietary supplement’s utility. Thus, creatine supplementation remains a potential therapy for Huntington’s disease, however further studies are needed.

### Amyotrophic lateral sclerosis

Amyotrophic lateral sclerosis (ALS), or Lou Gehrig’s disease, is marked by the loss of voluntary muscle control from the progressive degeneration of motor neurons ^78, 79^ with subsequent neuronal loss resulting in paralysis ^80, 81^. The cause and cure for ALS remain elusive. The most promising treatment is the drug riluzole, which only increases the lifespan of those with the disease by 6 months ^82^. To complicate the search for effective treatments, as many as 50% of ALS patients experience cognitive impairment that is revealed when they undergo specialized testing for neuropsychological deficits ^83^. In addition to motor neuron loss observed in ALS patients, cognitive impairment most often associated with the frontotemporal region of the brain is not always present ^83^. Furthermore, despite identifying a genetic overlap in ALS and other neurodegenerative disease mechanisms, the sporadic occurrence of cognitive impairment in ALS patients obscures understanding a clear etiology of the disease ^83^.

At a molecular level, ALS is characterized by altered glutamate homeostasis, oxidative damage, elevated intracellular calcium concentrations, mitochondrial swelling, and electron transport chain complex I deficiencies leading to reduced energy intake ^84– 87^. Generally, mutations in the gene responsible for the production of the enzyme, superoxide dismutase (SOD1) are common in many cases of familial ALS ^80^. The loss of function associated with SOD1 mutations reported in ALS translates to the accumulation of toxic free radicals from superoxide generated by the mitochondria ^80, 88, 89^. This suggests that the build up of free radicals results in altered energy production. Thus, creatine may serve as an energy alternative that is beneficial for ALS patients.

Klivenyi and colleagues studied transgenic mice with a mutated human SOD1 gene and assessed the neuroprotective effects of creatine. This was in response to the promotion of survival and improved motor coordination they observed with long-term creatine supplementation ^90^. Along with the proposed creatine benefits in protecting neurons from insufficient energy production, the results indicated that creatine administration protected neurons from oxidative damage. In contrast, two completed clinical trials in 2003 and 2004 tested oral creatine supplementation and provided little notable improvements in lifespan, muscle strength, or motor unit numbers in patients with ALS ^91, 92^. Although there is an observed trend toward enhanced survival following creatine supplementation, these studies distinguished between large differences (30–50% difference). Due to the high threshold for observing significant differences, there remains a possiblilty that creatine has a subtle effect. Currently, the long-term effects of creatine supplementation are being studied in a phase II clinical trial associated with the Northeast Amyotrophic Lateral Sclerosis Consortium (NEALS).

### Long-term memory

The ability to encode new memories (working memory), recall previous events (episodic/long-term/declarative memory) and short-term (primary/active) memory declines with age ^93, 94^. Previous reports indicated that phosphocreatine stores are quickly depleted upon brain activation, while ATP concentrations remain constant ^95, 96^. A reduction in creatine levels may have an effect on the immediate recall of knowledge based on the dramatic drop in phosphocreatine levels from brain activation. In 2003, Rae and colleagues questioned if creatine supplementation enhanced intelligence in healthy subjects ^97^. For 6 weeks, subjects were given 5 grams of creatine orally per day. Following this protocol, the subjects showed improvements in working memory and intelligence utilizing the backward digit span and Raven’s Advanced Progressive Matrices tasks, respectively. Furthermore, each of the participants were vegetarians, which supported the role that exogenous creatine has on increasing participants’ serum creatine levels. Using magnetic resonance spectroscopy to measure creatine levels, it was determined that creatine levels increased in the brain, solidifying that creatine is not solely distributed in skeletal muscle ^98^. Creatine supplementation has been considered for the improvement of memory in the elderly. One such study in the United Kingdom reported that subjects with an average age of 76 saw improvements in long-term memory when supplemented with 20 grams per day of creatine for 1 week ^99^. In addition to improvement in the long-term memory task, the elderly subjects improved in both forward and backward spatial recall as well as forward number recall. Based on these results, the investigators concluded that creatine could enhance cognition in elderly subjects, although follow up studies have not elucidated a mechanism by which creatine does this. Currently, there are few studies focused on the role that creatine supplementation plays on cognition and memory. In addition, *how* creatine improves memory in the aforementioned studies is yet to be understood at the molecular and cellular levels.

### Alzheimer’s disease

Alzheimer’s disease (AD) is a neurodegenerative disease marked by neurofibrillary plaques and tangles in the brain. One of the challenging characteristics of AD is the inability to definitively determine if a patient has the disease while alive. Only during the postmortem exam the disease can be definitively diagnosed ^100^. However, these hallmarks can be observed in the postmortem brains of individuals that did not display dementia or deteriorated cognitive function. As the disease progresses, the symptoms include severe dementia, confusion, and the loss of long-term memory. Although the onset of AD is not entirely understood, studies have shown that high-energy metabolism precedes the onset of AD while there are increased levels of myo-inositol, an important structural component of lipids, in comparison to the relative creatine concentrations. Furthermore, this increase in the ratio of myo-inositol and creatine precedes the onset of dementia in individuals with Down’s syndrome ^101– 103^. Creatine was shown to be protective of rat hippocampal neurons when confronted with beta-amyloid (Aβ) toxicity ^104^. One possible mechanism to intervene with the progression of Alzheimer’s disease is creatine kinase. Creatine kinase is responsible for the conversion of ATP to ADP and vice versa ^105^ and tends to be susceptible to high levels of oxidative damage in the brains of Alzheimer’s disease patients ^105^. In Alzheimer’s disease, creatine kinase activity is reduced by as much as 86% along with a reduction of in creatine kinase protein expression of 14%, which suggests that the Alzheimer’s disease brain has lower levels of phosphocreatine in the beginning stages of the disease ^101, 106^. Although creatine kinase levels were reduced, studies have questioned the involvement of creatine upon finding deposits of the molecule in amyloid precursor protein (APP) in transgenic mice ^107^. The solubility of creatine in an aqueous solution is 100 mM, however the total creatine concentration in the brain only reaches 20 mM ^10^. Possible explanations for the origin of these creatine deposits in the transgenic mice models include: (1) spillage from neuronal cell death, (2) excess oligodendrocyte production of creatine, (3) limited creatine uptake by the CRT and (4) the oxidation of creatine kinase that limits the formation of phosphocreatine ^108– 111^. Although each of these possibilities has been studied extensively, it remains unclear to the exact origin of the creatine deposits. This further reiterates that the exact role of creatine in AD is still yet to be understood and may be more complicated than previously thought.

### Stroke

Stroke is “defined as an acute neurologic dysfunction of vascular origin with sudden (within seconds) or a least rapid (within hours) occurrence of symptoms and signs corresponding to the involvement of focal areas in the brain” ^112^. In the past decade, stroke has been the second leading cause of death ^113^. Ischemic stroke, which is more common ^114, 115^, occurs when the brain is deprived of glucose and oxygen due to insufficient blood supply. This causes acidosis, an increase in intracellular calcium, and the formation of reactive oxygen species leading to ischemic cell death ^116^. Symptoms of ischemic stroke include sudden numbness on one side of the body, vision disturbances, difficulty in speaking and understanding, imbalance, and loss of coordination. The available treatments for ischemic stroke consist of the use of tissue plasminogen activator (tPA) ^117^ and/or surgical treatments. The commonly used *in vitro* model for ischemic stroke involves oxygen-glucose deprivation ^118, 119^ and rodent model for stroke involves mechanical or thromboembolic occlusion of cerebral vessel to mimic cerebral ischemia ^120– 123^.

An early study involving eight stroke patients has demonstrated that creatine and phosphocreatine content is reduced in the ischemic brain ^124^. In rat hippocampal slices, pre-incubation with creatine (0.03–3 mmol/L) dose-dependently reduced damage due to anoxia ^125^. Another *in-vitro* study in rat hippocampal slices showed dose dependent increase in phosphocreatine concentration and delay in anoxic depolarization after incubation with 1 mM creatine ^126^. Creatine was shown to exert protective antioxidant effect in U937 human promonocytic cells after oxidative damage ^127^. Creatine has been shown to be neuroprotective in an experimental model of anoxia in neonatal mice supplemented with creatine ^128– 130^. Also, rats subjected to creatine pretreatment before cerebral hypoxia showed a reduction (25%) in the volume of edematous brain tissue compared when compared to control ^131^. Zhu *et al.* showed that mice supplemented with 2% creatine in the diet showed reduced neuronal damage compared to control groups following middle cerebral artery occlusion that causes ischemic stroke. The study indicated that this beneficial effect of creatine is due to the restoration of energy depletion and inhibition of caspase activation along with some other unknown mechanisms ^132^. These studies support the fact that creatine could be a potential compound to be used as a prophylactic, or preventative, dietary supplement in patients at high risk for stroke ^133^. Still, more work is needed to demonstrate the efficacy of creatine to prevent stroke and to develop a creatine supplementation regimen to help patients at risk for stroke to avoid the debilitating event.

## Conclusions

Creatine has the potential to elicit positive effects in muscle strength, memory, and has further influence on neurodegenerative conditions. It remains to be seen if creatine has the ability to alter age-associated, progressive neurodegenerative disorders once individuals are in intermediate or late stages of the disease. However, based on the creatine interaction with energy metabolism and subsequent neuroprotective mechanisms, the interest for studying alternative uses for creatine in physiology is enhanced. Unfortunately, the phase III clinical trial for Parkinson’s disease and creatine was halted. However, there are ongoing clinical trials for creatine. Creatine supplementation is in a phase III clinical trial for the treatment of the energetic deficiencies in Huntington’s disease. With promising effects thus far, CREST-E remains continually funded by the National Center for Complementary and Alternative Medicine (NCCAM) and the Food and Drug Administration (FDA) as the largest therapeutic trial ever for Huntington’s disease. The usefulness of this compound may prove an important deterrent at beginning stages of dementia or even for increasing muscle strength in Parkinson’s disease patients. Furthermore, creatine may serve as a preventative treatment for the long-term consequences of stroke but may play a more complicated role in Alzheimer’s disease. Despite the wide range of uses for creatine supplementation, this dietary supplement should be the focus of additional studies for the treatment of age-related diseases. Going forward, one must consider if there are other mechanisms for which creatine acts to be protective and beneficial. These alternative mechanisms and the molecular/cellular targets for creatine remain to be determined and fully characterized.
