# Supplementary material for: A review of creatine supplementation in age-related diseases: more than a supplement for athletes
Source: F1000Res. 2014 Sep 15;3:222. [Version 1] doi: 10.12688/f1000research.5218.1 (PMC4304302; doi:10.12688/f1000research.5218.1)
Supplement: Peer review history

# Peer review: Referee response for version 1

**DOI:** 10.5256/f1000research.5564.r7059

**Published:** 2015-01-16

**Authors:** Paula Bickford

**Affiliations:** 1 Department of Neurosurgery and Brain Repair, University of South Florida College of Medicine, Tampa, FL, USA

This is an excellent review of the literature on creatine. I think that it would benefit from more discussion on possible side effects or toxicology. There are a number of review articles that discuss both the benefits and potential toxicology that could be included. 

Overall the article is well written and a comprehensive review of the literature. It is interesting that such diverse effects are being seen in a wide range of degenerative diseases.

I have read this submission. I believe that I have an appropriate level of expertise to confirm that it is of an acceptable scientific standard.

# Peer review: Referee response for version 1

**DOI:** 10.5256/f1000research.5564.r6140

**Published:** 2014-11-04

**Authors:** James Simpkins

**Affiliations:** 1 Department of Gastroenterology and Hepatology, West Virginia University Health Science Center, Baltimore, MD, 21205, USA

This is solid contribution to the literature on creatine and its possible beneficial role in acute and chronic neurodegenerative conditions.

I have one major comment and several minor issues:

Major: the manuscript does not, but should, deal to any extent with possible side-effects of creatine supplementation. Particularly since the population proposed for intervention are largely elderly and very ill.  A paragraph stating this and possible side-effects should suffice.

Minor things:

1. 'Parkinson’s disease' section, paragraph 2, sentence 7, please insert "not" before "results".
2. 'Amyotrophic lateral sclerosis' section, second paragraph, SOD-1 is cytosolic, so more than just mitochondrial proteins would be affected. This paragraph should be changed to reflect this.
3. 'Long-term memory' section, third sentence, I would replace the term "dramatic" with another term as little drama happens in results of studies.
4. 'Alzheimer's disease' section, first sentence - the phrase should read "amyloid-containing plaques and neurofibrillary tangles".
5. 'Alzheimer's disease' section - define CRT.

I have read this submission. I believe that I have an appropriate level of expertise to confirm that it is of an acceptable scientific standard.
